# Guidance for high-dose vitamin D supplementation for prolonging the honeymoon phase in children and adolescents with new-onset type 1 diabetes

**DOI:** 10.3389/fendo.2022.974196

**Published:** 2022-08-18

**Authors:** Benjamin Udoka Nwosu

**Affiliations:** ^1^ Division of Endocrinology, Department of Pediatrics, Zucker School of Medicine at Hofstra/Northwell, New Hyde Park, NY, United States; ^2^ Division of Pediatric Endocrinology, Department of Pediatrics, University of Massachusetts Medical School, Worcester, MA, United States

**Keywords:** vitamin D, honeymoon phase, partial clinical remission, insulin sensitivity, insulin dose adjusted A1c, hemoglobin A1c, type 1 diabetes

## Abstract

The publication of our recent randomized controlled trial (RCT) showing that vitamin D could protect the β-cells during the honeymoon phase of type 1 diabetes (T1D) has led to calls for guidance for vitamin D supplementation during the critical phase of type 1 diabetes. Prolonging the partial clinical remission (PR) phase of TID improves glycemic control and reduces long-term complications of T1D. This RCT randomized 36 children and adolescents to either receive vitamin D_2_ (ergocalciferol, given as 50,000 international units per week for 2 months and then every other week for 10 months) or a placebo. The results showed that vitamin D significantly decreased the temporal rise in both hemoglobin A1c at a mean rate of changes of 0.14% every 3 months versus 0.46% every 3 months for the placebo group (p=0.044); and in the functional marker of PR, the insulin-dose adjusted A1c at a mean rate of change of 0.30% every 3 months versus 0.77% every 3 months for the placebo group, (p=0.015). We recommend a baseline estimation of 25(OH)D concentration at the time of diagnosis of T1D, and to begin vitamin D supplementation if serum 25(OH)D concentration is <30 ng/mL, to maintain serum 25(OH)D concentrations between 30-60 ng/mL. If serum 25(OH)D concentration is >30 ng/mL, monitor vitamin D status with serial 25(OH)D estimations; and initiate vitamin D supplementation if serum 25(OH)D concentrations drop to <30 ng/mL. Continue vitamin D supplementation for at least one year to ensure optimal benefit from vitamin D supplementation during the partial clinical remission phase of type 1 diabetes.

## Introduction

Type 1 diabetes (T1D) is a chronic debilitating disease with an annual cost of $327 billion in the U.S (1). T1D affects 1.6 million Americans, of which 187,000 are children and adolescents (2). The pathogenesis of T1D stems from the autoimmune destruction of the β-cells of the pancreas leading to insulinopenia and persistent hyperglycemia (3). At the time of diagnosis of T1D, up to 50% of pancreatic β-cell may remain viable and this residual β-cell function (RBCF) may persist for months or years (4–6). This is called the partial clinical remission (PR) phase or the honeymoon phase of T1D. The PR is a critical phase where the natural history of T1D could be positively modified to reduce the burden of the disease. Prolonging the duration of PR improves glycemic control and reduces long-term complications of T1D (7–10). Several attempts have been made in the past few decades to prolong the duration of the honeymoon phase (11). Recent attempts using immunomodulatory and immunosuppressive agents to stop the destruction of β-cells have shown some promise, but the protection is short-lived and insufficient (11–14). For example, Teplizumab, an anti-CD3 monoclonal antibody, appears to act on the preclinical phase of T1D by reducing β-cell inflammation, however, its pharmacodynamic properties preclude its widespread acceptability (12). These include several side effects such as headache, nausea, decreased white blood cell count, and severe rashes. Previous studies that used lower doses of vitamin D had reported unclear results on vitamin D’s impact on PR (8, 15), thus suggesting that high-dose vitamin D may be necessary to detect vitamin D’s extra-skeletal effects in humans (11, 16) as has been reported in animal studies (17). Pozzilli and co-workers (18) hypothesized that vitamin D has immunomodulatory functions that favor Th2 immune response that could protect residual β-cells from further destruction following the diagnosis of T1D. Our recently concluded randomized controlled trial (RCT) demonstrated that vitamin D is safe in children, and its immunomodulatory and anti-inflammatory actions could protect RBCF (18, 19), thus suggesting that adjunctive therapy with an inexpensive and easily available vitamin D could increase RBCF and lengthen PR (8, 18) **(** **Table 1** **)**. We hypothesized that adjunctive ergocalciferol supplementation would increase RBCF and prolong PR. The study’s primary aim was to investigate the effect of ergocalciferol on RBCF and PR in children and adolescents with T1D. The primary outcome was the change over time in RBCF as measured by stimulated C-peptide (SCP) and the insulin-dose adjusted A1c (IDAA1c).

**Table 1:** Summary of the Temporal Trends in Hemoglobin A1c and the Insulin Dose Adjusted Hemoglobin A1c during the trial.

Parameter	Vitamin D (ergocalciferol)	Placebo	P value
Hemoglobin A1c (%)	0.14	0.46	0.044
Insulin-dose adjusted hemoglobin A1c (%)	0.30	0.77	0.015

This RCT was the first study to demonstrate significant differences in functional and dynamic parameters between an ergocalciferol treatment group and a placebo group as shown by ergocalciferol’s ability to significantly reduce the rise in the rates of change of both the hemoglobin A1c (HbA1c), and the IDAA1c, the functional marker of PR. IDAA1c is a two-dimensional functional marker of PR that integrates both the total daily dose of insulin and HbA1c as parameters in the prediction of residual β-cell function (20). As a result of the strong correlation between IDAA1c and stimulated C-peptide level of >300 pmol/L (21), the International Society for Pediatric and Adolescent Diabetes recommends IDAA1c as its gold standard test for PR (22, 23). Our RCT is also the longest investigation in PR in an exclusively pediatric population receiving standardized insulin regimen and high dose ergocalciferol. The dose of ergocalciferol used in this study was similar to the dose used in other studies that suggested that higher doses of vitamin D are necessary for the detection of its extra skeletal functions (11, 16). The patients’ characteristics and the RCT design make the results of the study generalizable to all youth with newly diagnosed T1D given the ethnic and racial diversity, the age range, and the duration of the trial. The rationale for this mini review was to publish some guidance for vitamin D supplementation in patients with newly diagnosed T1D.

## Summary of methods and results

The details of the methods and results of our RCT are published elsewhere (19). As noted in our primary publication, we randomized 36 participants of 10-21 years who were recently diagnosed with T1D in a 12-month randomized, double-blind, placebo-controlled trial of ergocalciferol versus placebo to investigate the effect of ergocalciferol on RBCF and PR in children and adolescents with new-onset T1D. Specifically, each subject received either 50,000 international units of ergocalciferol once weekly for 2 months, and then every other week for 10 months, or placebo. Glycemic control was standardized using treat-to-target insulin regimen.

The results showed that both groups were comparable at study entry. There was a significant rise in serum 25(OH)D concentration in the ergocalciferol group compared to the placebo group. Serum TNF-α was significantly lower in the ergocalciferol arm compared to the placebo. The ergocalciferol group demonstrated a significant blunting of the temporal rise in both their A1c and the IDAA1c, the functional marker of PR **(** **Table 1** **)**.

## Mechanism of β-cell protection by high-dose vitamin D

Vitamin D has anti-inflammatory and immunomodulatory properties (11, 24). Studies in animals showed that vitamin D prevents insulitis by downregulating pro-inflammatory chemokine production by β-cells (25) through its immunomodulatory activities and direct effect on β-cell function (26). Pozzilli et al (18) hypothesized that vitamin D has immunomodulatory functions that favor Th2 immune response that could protect residual β-cells from insulitis. Our RCT reported a significant reduction in the serum concentration of the pro-inflammatory agent (27), TNF-α, in the ergocalciferol group versus the placebo. This suggests that vitamin D could lower inflammation and insulitis during the honeymoon phase of T1D by decreasing serum TNF-α concentrations. Our results further suggest that vitamin D lowers insulin resistance as depicted by the significant blunting of the trajectories of the increases in both A1c and IDAA1c suggesting that vitamin D supplementation slowed increased insulin requirements by increasing insulin sensitivity in patients with new-onset T1D.

## Guidance for high-dose vitamin D supplementation in patients with newly diagnosed type 1 diabetes

**Figure 1:**
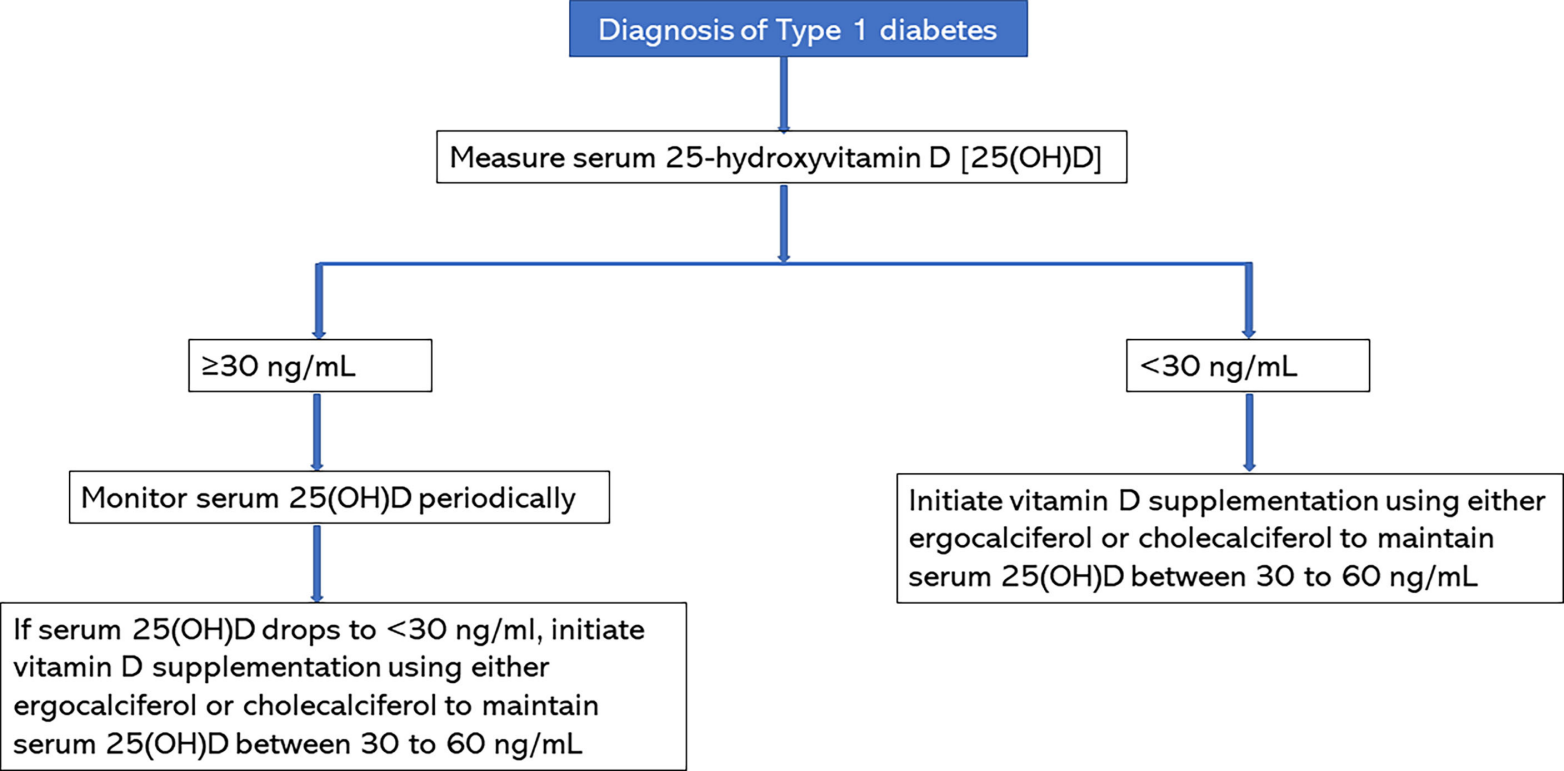
Protocol for vitamin D supplementation in patients with new-onset type 1 diabetes.

Baseline 25(OH)D concentration should be assessed in all patients with new-onset T1D. As shown in **Figure 1**, subjects with serum 25(OH)D concentrations of <30 ng/mL should receive vitamin D supplementation with either ergocalciferol or cholecalciferol to maintain serum 25(OH)D concentrations >30 ng/mL and below the upper limit of the assay. Based on our study, a serum 25(OH)D target of 30-60 ng/mL is safe and effective (19). Patients with serum 25(OH)D concentrations of >30 ng/mL should be monitored with serial 25(OH)D concentrations and should be started on vitamin D supplementation if serum 25(OH)D concentrations drop to <30 ng/mL. The vitamin D supplementation regimen should be continued for at least one year (28) to ensure optimal benefit from vitamin D during the partial clinical remission phase of type 1 diabetes. Our RCT showed that a dose of 50,000 international units per week for 2 months, and then every other week for 10 months was safe, well tolerated with no side effects in children and adolescents of ages 10-21 years. There was no case of vitamin D toxicity, hypercalcemia, or hypercalciuria. Therefore, physicians should feel comfortable initiating treatment in this age group according to our RCT regimen. For children <10 years of age, we recommend 1000 to 2000 IU per day to maintain serum 25(OH)D between 30-60 ng/mL.

In conclusion, the systematization of the protocol and guidance for adjunctive high-dose vitamin D supplementation in patients with new-onset T1D using data from RCTs will ensure accountability for rigorous approach and patient safety, while protecting the surviving β-cells. This has the potential to positively alter the natural course of T1D and may reduce the burden of the complications of the disease.

## Author contributions

The author confirms being the sole contributor of this work and has approved it for publication.

## Funding

This study was funded in part by an investigator-initiated research grant, Grant ID: 5 R21 DK113353-03, to BN from NIDDK, NIH

## Acknowledgments

We thank Professor Alan D. Rogol for his expert review of this manuscript.

## Conflict of interest

The author declares that the research was conducted in the absence of any commercial or financial relationships that could be construed as a potential conflict of interest.

## Publisher’s note

All claims expressed in this article are solely those of the authors and do not necessarily represent those of their affiliated organizations, or those of the publisher, the editors and the reviewers. Any product that may be evaluated in this article, or claim that may be made by its manufacturer, is not guaranteed or endorsed by the publisher.
